# Supplementary material for: The critical current of disordered superconductors near 0 K
Source: Nat Commun. 2020 May 29;11:2667. doi: 10.1038/s41467-020-16462-8 (PMC7260374; doi:10.1038/s41467-020-16462-8)
Supplement: Supplementary file 1 — Supplementary Information [file 41467_2020_16462_MOESM1_ESM.pdf]

Supplementary Information

The Critical Current of Disordered Superconductors near 0 K

Doron et al.

Supplementary Note 1. SAMPLE CHARACTERIZATION

In the main text we discuss measurements of three a:InO samples of different thicknesses (26, 100 and 280 nm). The study was actually performed on two more samples of thicknesses 22nm and 57nm. We did not include data of the 57nm thick film in the main-text only because we did not measure sufficiently detailed zero-bias $R(T)$'s of this sample to perform a heat-balance analysis. The 22 nm film did not show discontinuities at critical currents, only large non-linearities. We chose to leave the question of why this thinner film did not display a discontinuous response to a future publication. In order to properly compare between samples, each sample was thermally annealed post deposition to a room T resistivity (ρ) of 4 $\pm$0.2 mΩ·cm, which places them in the relatively low disorder range of a:InO. T_c of these samples is approximately 2.4K (measured only for the 26nm thick film due to technical reasons) and the width of the transition (90% to 10% of normal state) is approximately 350mK. The mean-free-path in our samples is close to 0.5nm (approximated using Drude's model and assuming a charge density typical to a:InO films of $5{\cdot}10^{20}$cm^{-3}) and the coherence length is $\xi \approx$5nm [1].

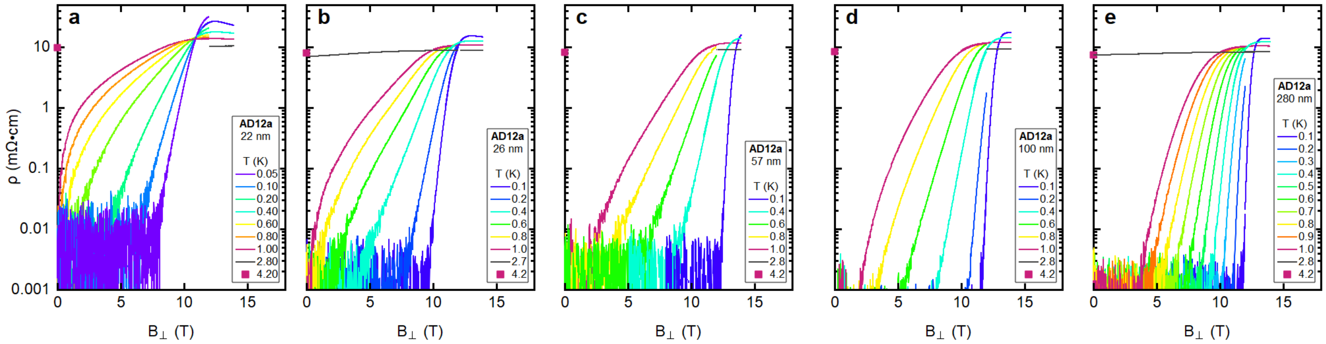

Supplementary Figure 1: **$\rho(B)$ of low-disordered samples of different thicknesses.** ρ (log scale) vs B of samples of thicknesses (a) 22nm (b) 26nm (c) 57nm (d) 100nm and (e) 280nm.

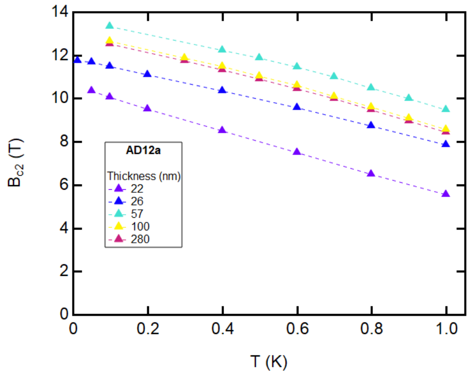

Supplementary Figure 2: **B_{c2} at low T.** B_{c2}, defined as B of half of the normal state R, vs. T of all five samples.

In Supplementary Figures 1a-e we plot $\rho(B_\perp)$ of each of the five samples where the color-coding marks different T's. In Supplementary Figure 2 we plot B_{c2} vs. T, extracted from Supplementary Figures 1a-e, of all five samples.

Supplementary Note 2. EXAMINING DE-PAIRING AS THE MECHANISM FOR J_c

Raising I through a superconductor increases the kinetic energy of a Cooper-pair. If the kinetic energy exceeds its binding energy (the superconducting gap) Cooper-pairs will break leading to a dissipative state. This dissipation mechanism is termed the de-pairing mechanism [2].

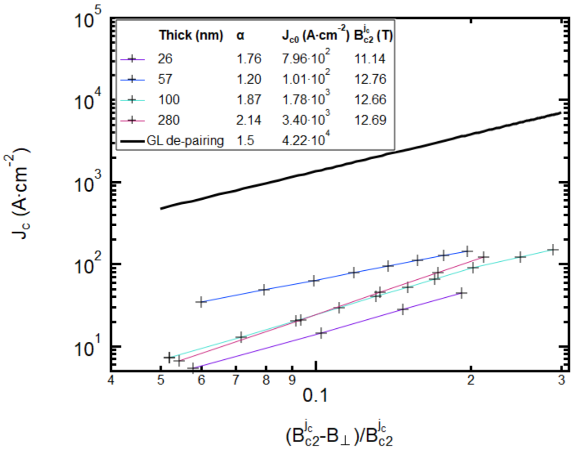

Supplementary Figure 3: ***B* dependence of J_c - A comparison to the de-pairing J_c.** J_c vs $\frac{B_{c2}^{j_c}-B}{B_{c2}^{j_c}}$ plotted for four samples of different thicknesses: 280nm (magenta), 100nm (turquoise), 57nm (blue) and 26 nm thick (purple). J_c can be fitted to a power-law according to Supplementary Equation (3). The values of the fit parameters for different samples appear in the figure. The black line marks the calculated de-pairing J_c (according to Supplementary Equation (2) and (1)).

The Ginzburg-Landau de-pairing J_c in SI units at $T \to 0$ and $B = 0$ is [2, 3]

$$J_{c0}^{GL} = \frac{\Phi_0}{3\sqrt{3}\pi\mu_0\lambda^2\xi} \tag{1}$$

where $\Phi_0 \approx 2.07 \cdot 10^{-15}$ T·m^2 is the magnetic flux quantum, λ is the London penetration depth, $\mu_0 = 4\pi \cdot 10^{-7}$ H/m is the vacuum permeability and ξ is the coherence length which is $\approx$ 5nm for a:InO samples [1]. One can estimate λ using the relation $\lambda^2 = \frac{tL_k}{\mu_0} = \frac{t\hbar^2}{\mu_0 e^2 \rho_{s0}}$ where L_k kinetic inductance t is the thickness, and ρ_{s0} is the superfluid stiffness at $B = 0$. From Ref. [4] we can extract for a t = 20nm thick a:InO film $L_K \approx$ 3nH (measured using a two-coil mutual inductance technique). Using Supplementary Equation (1) results in $J_{c0}^{GL} = 4.22 \cdot 10^4$A·cm^{-2}. From Ref. [5] we can extract for a t = 20nm thick a:InO film $\rho_{s0} = 8 \cdot K_B$ K (measured using ac conductivity measurements at 9-22GHz) where K_B is the Boltzman constant. Inserting that in Supplementary Equation (1) leads to a comparable result $J_{c0}^{GL} = 3.23 \cdot 10^4$A·cm^{-2}.

From Eq. 5 of Ref. [6] We can calculate the B dependence of J_c^{GL}

$$J_c^{GL}(B) = J_{c0}^{GL}(\delta B_{c2})^{3/2} \ ; \ \delta B_{c2} \equiv \frac{B_{c2} - B}{B_{c2}} \tag{2}$$

The resulting J_c vs δB_{c2} is plotted as the black line in Supplementary Figure 3.

Following the analysis of Ref. [6], it turns our that J_c of superconducting a:InO films (of a similar disorder level to the films studied in the main-text) also follows a similar power-law behavior which is described in Supplementary Equation (3)

$$J_c(B) = J_{c0}(\delta B_{c2}^{j_c})^{\alpha} \ ; \ \delta B_{c2}^{j_c} \equiv \frac{B_{c2}^{j_c} - B}{B_{c2}^{j_c}} \tag{3}$$

where J_{c0} and α and are fit parameters and $B_{c2}^{j_c}$ is set such that J_c will best fit a power-law in $\delta B_{c2}^{j_c}$ (it was shown in Ref. [6] that $B_{c2}^{j_c}$ is a good candidate for the definition of B_{c2}). In Supplementary Figure 3 we plot J_c vs $\delta B_{c2}^{j_c}$ for four of our films (26nm, 57nm, 100nm and 280nm thick films). The values of the fit parameters J_{c0} and α and B_{c2} for each sample are written in the inset of Supplementary Figure 3.

Although in both cases J_c has a power-law dependence there are significant differences between the measured J_c and the calculated de-pairing J_c: First, the calculated de-pairing J_{c0} is 10-400 times greater than J_{c0} we extract from the fit to Supplementary Equation (3). And second, α in the de-pairing description should be 3/2 [2, 6] where we measure a sample dependent α that assumes values between $1.2 - 2.14$.

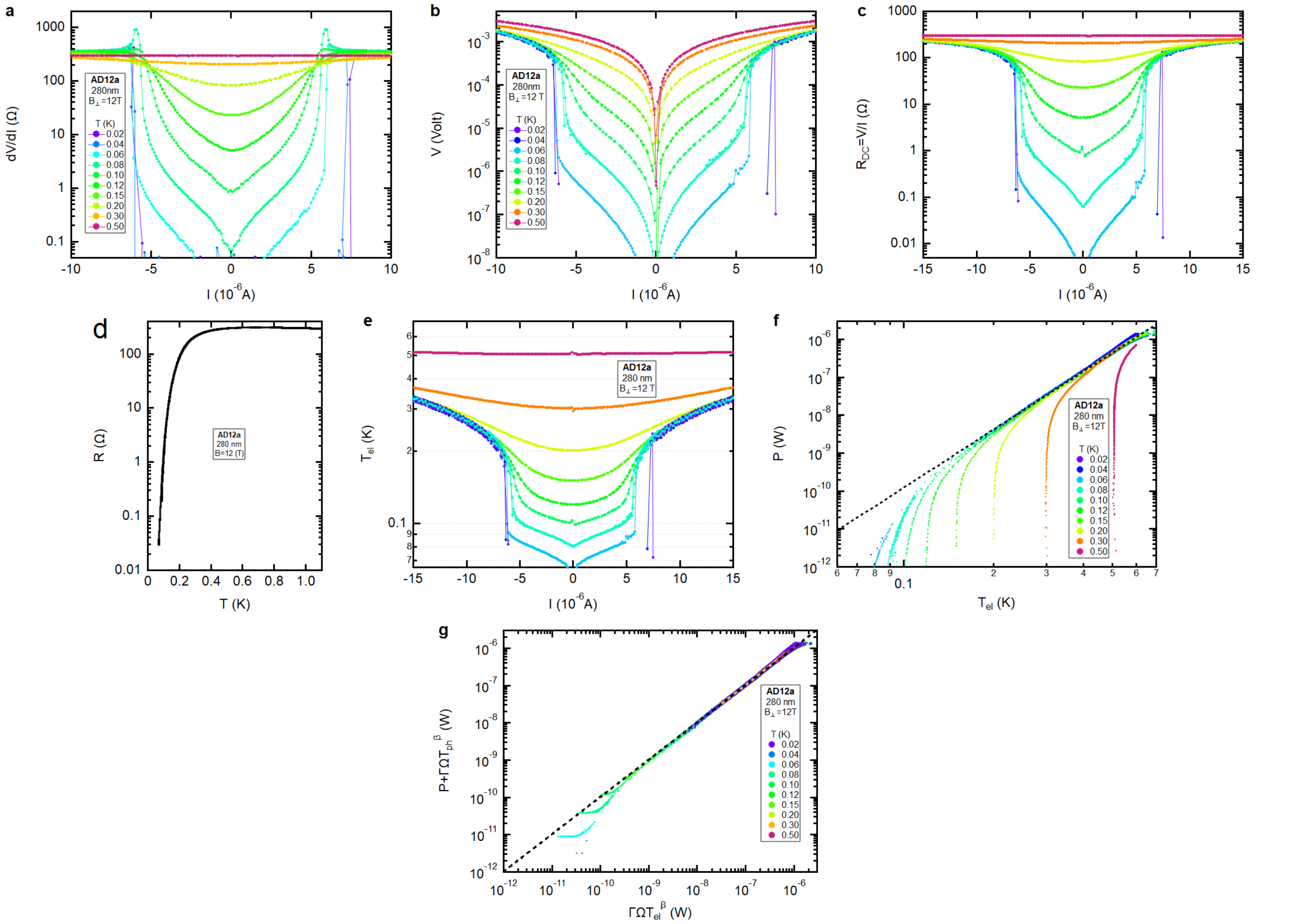

Supplementary Figure 4: Heat-balance analysis (a) $\frac{dV}{dI}$ vs I. (b) V vs I. (c) $R_{\mathrm{dc}} \equiv \frac{V}{I}$ vs I. (d) $R(I = 100\mathrm{nA})$ vs T. (e) T_{el} vs I. (f) P vs $\Gamma\Omega T_{\mathrm{el}}^{\beta}$. (g) $P + \Gamma\Omega T_0^{\beta}$ vs $\Gamma\Omega T_{\mathrm{el}}^{\beta}$.

One of the key findings in Ref. [6] is that the measured critical exponent α in three a:InO films is $1.62, 1.65, 1.67 \pm 0.02$ which they note is similar to $3/2$. Our results show that critical exponent α seems to be sample dependent. For a proper comparison between the findings of the two experiments one should note the following differences:

1. We defined I_c as $I_c^{\mathrm{H}\to\mathrm{L}}$ while in Ref. [6] I_c was defined as $I_c^{\mathrm{L}\to\mathrm{H}}$. As the hysteresis is very limited this difference in definition should not be significant.

2. To properly measure critical exponents one should have a scaling relation that spans over many orders of magnitude in the scaling parameter $\delta B_{c2}^{j_c}$. In Supplementary Figure 3 the scaling is only over a factor of 4-6 in $\delta B_{c2}^{j_c}$ and in Ref. [6] it spans over a slightly larger but still unremarkable factor of 10-20 in $\delta B_{c2}^{j_c}$.

3. In Ref. [6] α is extracted for samples of a single thickness of 30nm. Here α is extracted for samples of various thicknesses. Note that although our extracted α is not monotonic in the thickness, α of the 26nm thick film is 1.76 which is not significantly different than the 30nm films of Ref. [6].

Supplementary Note 3. DETAILED DESCRIPTION OF THE HEAT-BALANCE FIT

In the main-text we present the results of a heat-balance analysis we performed in order to explain the discontinuities observed at J_c. Here we present a detailed step-by-step account of this analysis.

In Supplementary Figure 4a we plot $\frac{dV}{dI}$ vs I of the 280nm thick film at $B_\perp = 12\mathrm{T}$ where the color-coding marks different T's. This measurement was performed in a 4-terminal configuration where in addition to the dc current

(x-axis) we also passed a 100nA low frequency (19.19Hz) ac current (we scaled $I_{\rm ac}$ for different samples according to the sample thickness maintaining a constant current density of $J_{\rm ac} \approx 0.1$ A·cm^{-2}). We simultaneously measured both the resulting ac V, from which we plot $\frac{dV}{dI}$ vs. I in Supplementary Figure 4a and the dc V. In Supplementary Figure 4b we plot the dc V vs I where V is extracted by a I-integration of the ac $\frac{dV}{dI}$ and is consistent with the measured dc V. For the heat-balance analysis we are interested in the dc measurement. The reason for that is two-fold: First, as the dc I's and V's are significantly larger than the ac component (by the design of our measurement) the power dissipated at the sample is $P \sim I_{\rm dc}V_{\rm dc}$. Second, one of the main assumptions of the heat-balance analysis is that all non-linear effects in the dc $I - V$'s originate from an elevated $T_{\rm el}$.

The next step is to extract $T_{\rm el}$ from the $V - I$ data of Supplementary Figure 4b [7–9]. In Supplementary Figure 4c we plot $R_{\rm dc} \equiv V/I$ vs I extracted from Supplementary Figure 4b. In the absence of electron-heating the linearity assumption would result in a constant $R_{\rm dc}$ (as observed in the $T = 500$mK magenta data). In Supplementary Figure 4d we plot a zero-bias $R(T)$ measurement where $I_{\rm dc} = 0$ and $I_{\rm ac} = 100$nA. As $I_{\rm ac} \ll I_{\rm c}$ and as a reduction of $I_{\rm ac}$ to 10nA did not change the value of R we assume that this measurement was performed in the linear regime therefore we can assume that $T_{\rm el}$ is equal to T of our dilution refrigerator and use this zero-bias measurement as a calibrated electron thermometer for the data of Supplementary Figure 4c. For example $R_{\rm dc}$ of 1 and 10Ω in Supplementary Figure 4c can be translated using the electron thermometry measurement of Supplementary Figure 4d to 100 and 130 mK respectively. The result of this process is plotted in Supplementary Figure 4e where we plot $T_{\rm el}$ vs I (data of $T_{\rm el} < 90$ mK is achieved by an extrapolation of the $R(T)$ of Supplementary Figure 4d and is not significant to any of the conclusions of this work).

In Supplementary Figure 4f we plot P vs the resulting $T_{\rm el}$ on a log-log scale where the color-coding marks different T's. When P is sufficiently large, $T_{\rm el}$ is much greater than the refrigerator's T and all isotherms coincide and follow a power-law. The dashed black line is a power-law fit from which we can extract $\beta = 5.1$ and $\Gamma = 0.595$ nW K$^{-\beta}\mu$m^{-3}. In table 1 we list the values of β and Γ for several samples at various B's in both superconducting and insulating samples. The dimensions of Γ are nW K$^{-\beta}\mu$m^{-3} which depend on β, therefore, in order to have a proper comparison between samples at different B's and different β we multiply Γ by $(1K)^{\beta}$. It is interesting that, although we are comparing samples on both sides of the disorder driven and B driven SIT's, both parameters β and Γ are always of the same orders of magnitude. These β and Γ are used for the graphical solution of the heat-balance equation from which we extract $J_{\rm c}$.

In Supplementary Figure 4g we plot $P + \Gamma\Omega T_0^{\beta}$ vs $\Gamma\Omega T_{\rm el}^{\beta}$ (as was done in Ref. [7, 10, 11]). Plotting the data that way we get that all isotherms coincide and data that fits the heat-balance equation falls on the dashed black diagonal line. This fit holds for over 4 orders of magnitude but one can see that there are deviations at low P's. There are several possible explanations for the origin of these deviations such as the model being oversimplified and that there is an accumulation of several errors in the translation of V to $T_{\rm el}$ which, at these low T's, become comparable to $\Gamma\Omega T_{\rm el}^{\beta}$. We discuss these deviations in Supplementary Note 6.

Supplementary Note 4. DEVIATIONS FROM OHMIC TRANSPORT IN THE LOW RESISTIVE STATE

As stated in the main-text, although the heat-balance analysis provides an accurate prediction of $I_{\rm c}$, there are notable deviations from Ohmic transport in the LR state below $I_{\rm c}$. To demonstrate the extent of these deviations, in Supplementary Figure 5a we plot the measured V vs I (magenta) at $B = 12$T and $T = 60$mK and in blue we plot the simulated s-shaped $V - I$ solution of the heat-balance equation using the heat-balance parameters extracted in Supplementary Note 3. $I_{\rm p}$ (see Supplementary Note 7) is marked by a dashed blue line. We see that although $I_{\rm c} \approx I_{\rm c}^{\rm min} \approx I_{\rm p}$ and both curves converge at the HR state, there are notable deviations at low V's. In Supplementary Figure 5b we plot $T_{\rm el}$ extracted from the measured data (magenta) and the simulated s-shaped $T_{\rm el}$ vs. I. It can be seen that the large deviations in V in Supplementary Figure 5a are a result of some added heating in the LR state that the heat-balance model does not predict. As discussed in the main-text, we consider these deviations to be a failure of the Ohmic assumption and a full account of these deviations awaits a theory that combines intrinsic non-Ohmic effects and self-heating.

Supplementary Note 5. MEASUREMENT OF THE KAPITZA RESISTANCE

Eq. 1 of the main-text describes the heat-balance between the electrons and phonons of the a:InO film. As mentioned in the main-text, we chose to assume that $\tilde{R}_{\text{el-ph}}$ is the largest $\tilde{R}$ and to write Eq. 1 in terms $T_{\rm el}$ and $T_{\rm ph}$ at the outset only for clarity and readability purposes. In fact, as the form of the Eq. 1 is general and describes various heat transfer

Sample name	SC/INS	B [T]	β	Γ [nW $K^{-\beta}\mu m^{-3} \times 1K^{\beta}$]
AD12a 26nm	SC	$9.5_{\perp}$	6.3	3.280
AD12a 26nm	SC	$10_{\perp}$	5.7	1.990
AD12a 26nm	SC	$11.25_{\|\|}$	5.8	1.990
AD12a 26nm	SC	$11.5_{\|\|}$	5.5	1.73
AD12a 100nm	SC	$10.5_{\perp}$	7.5	3.786
AD12a 100nm	SC	$11.5_{\|\|}$	7.5	3.786
AD12a 280nm	SC	$9.5_{\perp}$	9.8	4.018
AD12a 280nm	SC	$10_{\perp}$	6.6	3.053
AD12a 280nm	SC	$10.5_{\perp}$	6.56	1.506
AD12a 280nm	SC	$11_{\perp}$	6.15	1.314
AD12a 280nm	SC	$11.5_{\perp}$	5.6	0.948
AD12a 280nm	SC	$12_{\perp}$	5.1	0.595
AD12a 280nm	SC	$12_{\|\|}$	5.96	1.456
GR12H2a	INS	$3_{\perp}$	8.7	2.050
RAM005b	INS	$11_{\perp}$	6	1.850
TL40a	SC	$0.1_{\perp}$	7	0.832
TL40a	SC	$0.3_{\perp}$	5.52	0.355
TL40a	SC	$1_{\perp}$	5.61	0.736
TL40a	INS	$3_{\perp}$	5.15	0.803
TL40a	INS	$6_{\perp}$	5.51	1.640
TL40a	INS	$9_{\perp}$	5.51	2.395
TL40a	INS	$11_{\perp}$	5.26	2.448

Supplementary Table 1: **β and Γ of superconducting and insulating samples**

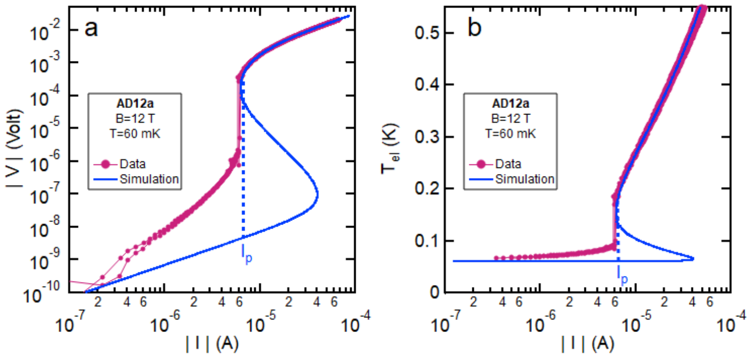

Supplementary Figure 5: **Comparison between measured and simulated solutions** (a) Measured V (magenta) and simulated s-shaped V vs. I of the 280nm thick sample at $B = 12$T and $T_{ph} = 60$mK. I_p (see Supplementary Note 7) is marked by a dashed blue line. (b) T_{el} extracted from measured data (magenta) and simulated s-shaped T_{el} vs. I.

mechanisms, the heat-balance analysis we performed and the extracted parameters β, $\Gamma\Omega$ and T_{el} remain valid even if the thermal bottleneck is between two other subsystems. Below we discuss the scenarios where the thermal bottleneck is between the substrate's phonons and the liquid helium (Kapitza resistance) and between the phonons of the host a:InO of the substrate.

First is the Kapitza resistance [12–15], where cooling is impeded due to an acoustic mismatch between phonons of the liquid helium and of the substrate. If that is the case $T_{el} \approx T_{ph} \approx T_{sub} > T_0$. To test this possibility we conducted an independent experiment and measured the Kapitza resistance of our substrate (a boron doped silicon wafer with $\rho < 5\text{m}\Omega\cdot\text{cm}$ with 580 nm thick oxide layer). The schematics of the sample appear in Supplementary Figure 6a

where all patterns where created using optical lithography and a:InO and Ti/Au contacts were prepared as detailed in the Methods. Carbon paint thermometers were prepared by first defining their geometry using optical lithography and then immersing the sample in carbon paint until it dries out. A representative $R(T)$ of thermometer T1 at different B's is plotted in Supplementary Figure 6b showing that the thermometer is insulating and B-independent. We emphasize that the thermometers are electrically disconnected from the each other and from the heater therefore heat flow is via the substrate. The thermometers are labeled T1, T2, T3 and T4 (T3 was broken) and the heater is labeled S0 according to the schematics.

In Supplementary Figure 6c we plot T measured at each thermometer vs the power dissipated at the heater P. It can be seen that the substrate indeed heats up at sufficiently high powers. Following Ref. [16] we can estimate the $T_{\rm sub}$ as

$$T_{\rm sub} = (\frac{P}{A\sigma} + T_0^4)^{1/4} \tag{4}$$

where A is the area of the substrate (5.7mm $\times$ 5.7mm for the Kapitza resistance experiment and 5.7mm $\times$ 1.9mm for sample AD12a of the main-text), T_0 is the dilution refrigerator's T and $\sigma = 50$ W·K^{-4}·m^{-2} (see Fig. 9.11 of Ref. [17]). The black dashed line marks $T_{\rm sub}$ calculate from Supplementary Equation (4) for the experimental parameters of our Kapitza resistance experiment. It can be seen that the theoretical description is in excellent agreement with our experimental data measured by thermometers T1, T2 and T4. We would like to emphasize that we did not use any fit parameters.

In Supplementary Figure 6d we plot the same data as in Supplementary Figure 6c and add (purple dots) $T_{\rm el}$ vs P of the 280nm thick sample at $T = 60$mK and $B_\perp = 12$T (the sample measured in the main-text). The continuous gray line marks $T_{\rm sub}$ calculate from Supplementary Equation (4) for the experimental parameters of sample AD12a at $B_\perp = 12$T and $T = 60$mK. It can be seen that although $T_{\rm sub}$ is elevated it still underestimates $T_{\rm el}$. For example, at a power of 10nW for the parameters of AD12a at $T_0 = 60$mK, $T_{\rm sub}$ is expected to be 75mK while $T_{\rm el} = 247$mK.

For completeness we can extract the Kapitza resistance of our experiment

$$\tilde{R}_{\rm K} \equiv \frac{\Delta T}{P} \tag{5}$$

In Supplementary Figure 6e we plot $\tilde{R}_{\rm K}$ vs T for thermometer T4. The dashed black line is $\tilde{R}_{\rm K} = R_0 T^{-3}$, a functional form used in the literature [15], where $R_0 = a/A$, A is the surface area (5.7×5.7mm^2) and $a = 0.02$ as was reported for the thermal boundary of materials with helium mixtures (see section 7.3.3 of Ref. [15]). This functional form, describing our measured $\tilde{R}_{\rm K}$, also has no fit parameters. This suggests again that the phenomenon measured here is indeed the increase in $T_{\rm sub}$ due to the P flowing across $\tilde{R}_{\rm K}$.

Another possibility is that the thermal bottleneck is between the phonons of the substrate and of a:InO [18]. In this scenario $T_{\rm el} \approx T_{\rm ph} > T_{\rm sub} \approx T_0$. We did not manage to rule out this possibility but we do view it as unlikely for two reasons; First, the thermal wavelength of the a:InO phonons at $T \approx 100$mK in greater than 1μm therefore larger than the sample's thickness (although not by orders of magnitude). Second, as plotted in Supplementary Figure 6f, P at the LR side of the discontinuity is highly B dependent where we do not expect the acoustic mismatch between the a:InO and the substrate (and between the substrate and the liquid helium) to have a noticeable B dependence.

Supplementary Note 6. RESPONSE TO THE ARGUMENTS MADE IN REF. [6] OPPOSING THE BI-STABILITY PICTURE

Much of the supplemental material of Ref. [6] is devoted to explaining why their $I_{\rm c}$'s, measured on a:InO superconducting samples of similar disorder strength to our, are not a result of a thermal bi-stability. They performed a heat-balance analysis as detailed in Supplementary Note 3 and in Refs. [7, 19] and plotted P vs $T_{\rm el}^{\beta} - T_{\rm ph}^{\beta}$ as displayed in Fig. S6b of the supplementary material of Ref. [6]. By assuming a power-law dependence, as in Eq. 1 of the main-text, they extract the parameters β and $\Gamma\Omega$.

In order to be consistent with the notations of the supplemental material of Ref. [6] and of Ref. [7], we sometimes refer in this discussion to the HR→LR transition as the re-trapping transition and the LR→HR transition as the escape transition.

Before diving into the details we would like to reiterate that electron-heating theoretical models are simplified models [19–23] with some simplified assumptions such as the Ohmic assumption. In fact, both in the superconducting data we present in the main-text and in the electron-heating description of the $I-V$'s in the insulating phase there are deviations at low P's (as noted and discussed in Refs. [7, 19]). Small deviations from these results are acceptable

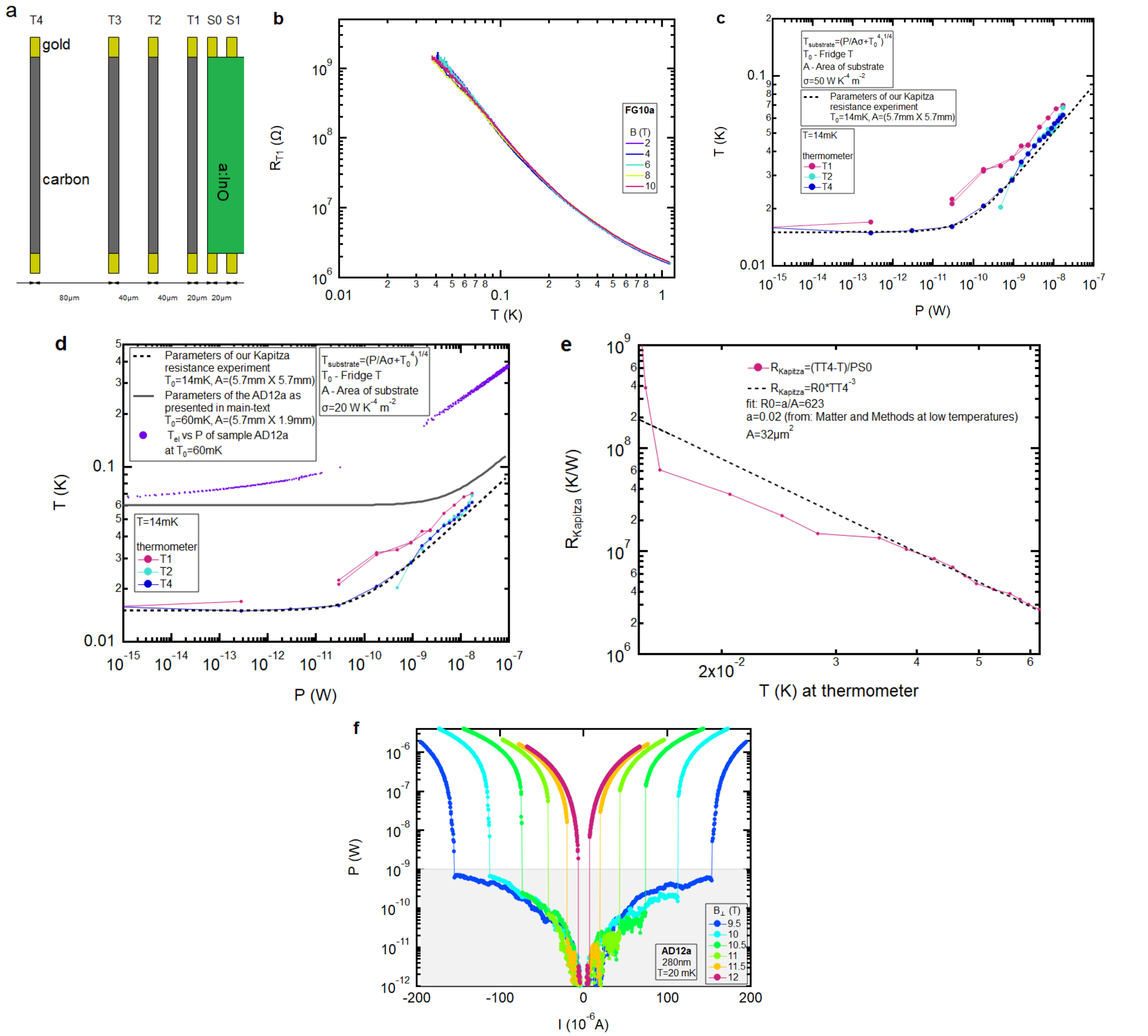

Supplementary Figure 6: **Measurement of the Kapitza resistance between the substrate and the helium mixture.** (a) Schematics of the sample. The yellow rectangles mark gold contacts, green marks a:InO (used as a heater) and gray marks carbon paint (used as thermometers). (b) R (log scale) vs T of a carbon paint thermometer. The color-coding mark different B's showing the thermometer is B-independent. (c) T measured at thermometers T1 (magenta), T2 (turquoise) and T4 (blue) vs Power dissipated at the heater at $T = 14$ mK. The dashed black line is the expected $T_{\rm sub}$ from Supplementary Equation (4) (with no fit parameters) for the parameters of our Kapitza resistance experiment. (d) The magenta, turquoise and blue data points and the dashed black line are the same as in Fig. (c). The purple data points are $T_{\rm el}$ vs P of the 280nm thick sample at $T = 60$mK and $B = 12$T (the sample measured in the main-text). The continuous gray line is $T_{\rm sub}$ from Supplementary Equation (4) for the parameters of the sample measured in the main-text. (e) $\tilde{R}_{\rm K}$ vs T at thermometer T4 where the magenta data points are extracted from Fig. (b) and the dashed black line is a fit achieved using parameters from the literature [15]. (f) P vs I of the 280nm thick sample at $T = 20$mK. The color-coding marks different $B_\perp$'s. The data in the gray portion of the figure is within the noise of the measurement. In contrast to what we observe, in the scenarios where the thermal bottleneck is between the substrate and either the liquid helium or the phonons of the a:InO P of the discontinuity should be independent of B.

within the over-heating framework and a full account of these deviations await a theory that incorporates self-heating and intrinsic non-Ohmic behavior.

One of the main claims of Ref [6] for rejecting thermal bi-stability as a cause for I_c is the scatter in the heat-balance analysis at low P's as displayed in Fig. S6b of the supplementary material of Ref. [6] for $P < 10^{-14}$W. We claim that, although these deviations can be a result of the over-simplified Ohmic assumption, they are also well within the error of the measurement as they are based on the low R data. This regime is extremely sensitive and any measurement error or noise will be amplified by the way the data is presented in Fig. S6b of the supplementary material of Ref. [6] as the x-axis should have very large error bars.

At low T's there will be a non-zero error in T_el (dT_el). This is because the translation to T_el is done by comparing R of both the zero-bias measurement and the $V - I$'s (see Supplementary Note 3) where at low T's both R's become exponentially small. At low I's we can assume that $T_\mathrm{el} \sim T_\mathrm{ph}$ therefore an error of dT_el will translate to $T_\mathrm{el}^\beta - T_\mathrm{ph}^\beta$ (x-axis of of Fig. S6b of the supplementary material of Ref. [6]) as:

$$\lim_{T_\mathrm{el}\to T_\mathrm{ph}} T_\mathrm{el}^\beta - T_\mathrm{ph}^\beta \approx \beta T_\mathrm{ph}^{\beta-1} dT_\mathrm{el} \tag{6}$$

where we assumed $T_\mathrm{el} = T_\mathrm{ph} + dT_\mathrm{el}$ and $dT_\mathrm{el} \ll T_\mathrm{ph}, T_\mathrm{el}$.

Inserting $\beta = 5.5$ and approximating $dT_\mathrm{el} \approx 3$mK (we estimate $dT_\mathrm{el} \sim 3$mK from Figs. S3 and S5b of the supplemental material of Ref. [6]. Although the zero bias $R(T)$ they present has an impressive activated behavior, there are still almost unavoidable deviations that are manifested in the noise in R at low T in their Fig. S3.) for $T_\mathrm{ph} = 70, 80, 100, 130$mK respectively ($\beta$ and T_ph of their data) results in error bars in the x-axis of $10^{-7}, 2{\cdot}10^{-7}, 5{\cdot}10^{-7}$ and $1.7 \cdot 10^{-6} K^{5.5}$. For all these T_ph's such an error bar deems the scatter at low P as insignificant.

A second claim made in the supplemental material of Ref. [6] is that, comparing to the results of the insulator [7] and the electron-heating theory of the insulator [19], the T dependence of the LR $\to$ HR switching current, $I_\mathrm{c}^{\mathrm{L}\to\mathrm{H}}$ is too weak, this is because they claim that in their heat-balance simulation $I_\mathrm{c}^{\mathrm{L}\to\mathrm{H}}$ diverges at low T's while their data shows that it does not diverge. This claim on their behalf is incorrect on both the theoretical and the experimental levels. The quantity they actually present is not $I_\mathrm{c}^{\mathrm{L}\to\mathrm{H}}$ but $I_\mathrm{c}^\mathrm{max}$. This is because, as discussed in the main-text, the heat-balance analysis [19] only predicts the limits of stability $I_\mathrm{c}^\mathrm{min}$ and $I_\mathrm{c}^\mathrm{max}$, and it does not attempt to predict where within this range the jumps will occur (as is stated in the work by Altshuler et al. "As it is usual for the first order phase transition the voltages, where the switches between HR and LR states happen (V_HL for HR $\to$ LR and V_LH for LR $\to$ HR switches), are determined by kinetics of the decay of metastable states. Theoretical analysis of this decay and evaluation of $V_\mathrm{HL,LH}$ is beyond the scope of this Letter. Here we can predict only their bounds"). Experimentally, in the insulating phase, we typically see that V_esc (V where the there sample switches from the low T_el solution to the high T_el solution) initially increases while cooling (much slower than $V_\mathrm{c}^\mathrm{max}$, the maximal V_esc predicted by the theory, which the equivalent of what is plotted in the simulation of the supplementary material of Ref. [6]). At very low T's, not only that it does not increase but it can decrease and saturate at a value similar to V_trap (V where the there sample switches from the high T_el solution to the low T_el solution). This can be seen in the Fig. 3 of Ref. [24] where this phenomenon is discussed in details. As shown in discussion section of the main-text and in Supplementary Note 7 of this supplemental material, the actual transition is consistent with a propagation of a switching wave that results in $I_\mathrm{p} \sim I_\mathrm{c}^\mathrm{min}$.

A third claim made in the supplemental material of Ref. [6] is regarding the B-dependence of the thermal bi-stability. While calculating the expected HR$\to$LR re-trapping I's from the heat-balance model at different B's they got that $I_\mathrm{c}^{\mathrm{H}\to\mathrm{L}}$ should act as a power-law in $|B_{c2}^{j_c} - B|^\alpha$ with a power of $\alpha = 2$. This they write is inconsistent with their measured $\alpha \geq 1.6$. As discussed above, in Supplementary Figure 3 we present a similar analysis for several samples and show that the value of this exponent α in non-universal, sample dependent, and can exceed 2.

A forth claim made in the supplemental material of Ref. [6] is that in some of the B's they measure a T dependence in $I_\mathrm{c}^{\mathrm{H}\to\mathrm{L}}$ while in the over-heating picture in the insulator the re-trapping was typically T-independent. As the theory does not predict the exact value of the critical currents it also does not prohibit a T-dependence of the re-trapping current. It does predicts that the lower limit of stability can only have a small T dependence. Having said that, from our vast experience with electron-heating in the insulating phase the re-trapping voltage is indeed typically T independent. But also in that insulating phase (where the authors of Ref. [6] write that "The hysteresis and current jump have been proven to be a direct consequence of a thermal bi-stability of the electronic system driven by Joule overheating") this is not always the case as we do sometimes observe T-dependent re-trapping currents (as displayed in Supplementary Figure 7a).

Their fifth and final claim is their most interesting claim where they point out that their measured dV/dI vs I at low I's is exponential ($\ln(dV/dI) \propto I$), which is consistent with vortex creep below the critical current, while in the heating scenario their simulation shows that $\ln(dV/dI) \propto I^2$. This is a very interesting claim that we do not have

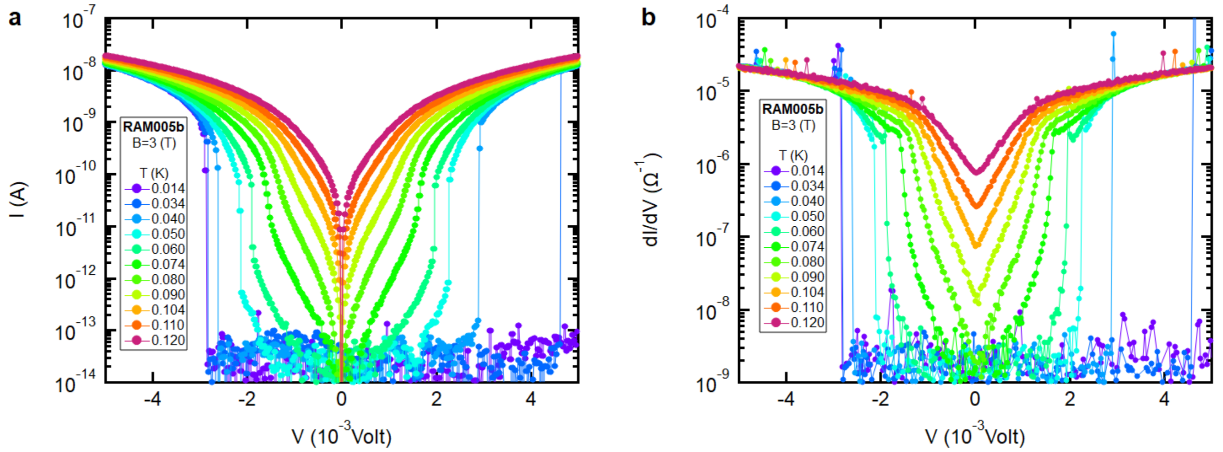

Supplementary Figure 7: (a) I vs V of sample RAM005b at $B = 3$T (in the insulating phase). The color-coding mark different isotherms. (b) dI/dV vs V of sample RAM005b at $B = 3$T (in the insulating phase). The color-coding mark different isotherms.

an answer to and we do not see any reason for vortex creep to be absent. As discussed in the main-text, such vortex creep is intrinsically non-linear as $\ln(dV/dI) \propto I$ and therefore its expected contribution does seems to be in contrast to one of the central assumptions of the heat-balance model [19] which assumes that any non-linearity is a result of an increase in $T_{\rm el}$.

In the supplemental material of Ref. [6] $\ln(dV/dI) \propto I$ vs $\ln(dV/dI) \propto I^2$ is used as a differentiating criteria between the phenomenon observed in the insulating phase of a:InO and the discontinuities observed in the superconducting phase of a:InO. We do not think that this is a good differentiating criteria. For example, in Fig. S2 of the supplemental material of Ref. [6] some of the data indeed behaves as $\ln(dV/dI) \propto I$ over some range but a significant portion of their measurements seem to better fit $\ln(dV/dI) \propto I^2$ (see Fig. S2 of their supplementary material, $B = 10.5$ at $T > 120$mK, $B = 11$T at $T > 100$mK, $B = 11.15$T at $T > 80$mK, $B = 11.4$T at $T > 60$mK and $B = 11.5$T at $T > 50$mK). We note that the $\propto I^2$ in their data is mostly at T's where the jump begins to diminish but these T's are still much smaller than the typical activation T which they relate to thermally assisted flux-flow (for example, in Fig S3 they show that at $B = 11.25$T the activation T is 0.75K where in Fig. S2 at $B = 11.4$T and $T \geq 69$mK $\ln(dV/dI) \propto I^2$). This is consistent with our superconducting films where in both $B_\perp$ and $B_{||}$ we observe that $\ln(dV/dI)$ sometimes better fits $\propto I^2$ than $\propto I$. On the other hand in Supplementary Figure 7b we plot dV/dI vs V measured on sample RAM005b at $B = 3$T in the insulating phase where we see that although they claim that such discontinuities are due to electron heating, $\ln(dV/dI) \propto V$. This shows that the $\ln(dV/dI) \propto I$ vs $\ln(dV/dI) \propto I^2$ criteria is lacking.

We summarize that, as the Ohmic assumption is merely an approximation, some deviations at low P's are acceptable within the over-heating framework. Such deviations at low P's in the insulating phase of a:InO are presented and discussed in Refs. [7, 19]. Some of the claims made in Ref. [6] against the electron-heating model are focused on this low P regime. A full account of these deviations awaits a theory that combines self-heating and intrinsic non-Ohmic behavior.

Supplementary Note 7. LACK OF HYSTERESIS - PROPAGATING SWITCHING WAVES

As discussed in the main-text, In our measurements there is only a limited hysteresis in $I_{\rm c}$ where the measured $I_{\rm c}^{\rm H\to L} \approx I_{\rm c}^{\rm L\to H}$ are very similar to the theoretical lower bound of the bi-stable I interval $I_{\rm c}^{\rm min}$ (see Fig. 3 of the main-text).

In chapter 3 of Ref. [25] the authors discuss the propagation of a switching wave in bi-stable conductors. They write that "in most cases the transitions between these states (these phases)" [namely the cold and hot $T_{\rm el}$ phases] "are initiated by local perturbations, which result in the nucleation of a new phase, which then propagates to cover the whole specimen". They proceed to assume that in the sample there are domains at a T of T_1 and of $T_3 > T_1$ and calculate the velocity of the domain wall separating these phases as a function of I. They show that there is some $I_{\rm p}$ where for $I > I_{\rm p}$ the domain with $T = T_3$ will expand and for $I < I_{\rm p}$ this domain will collapse and

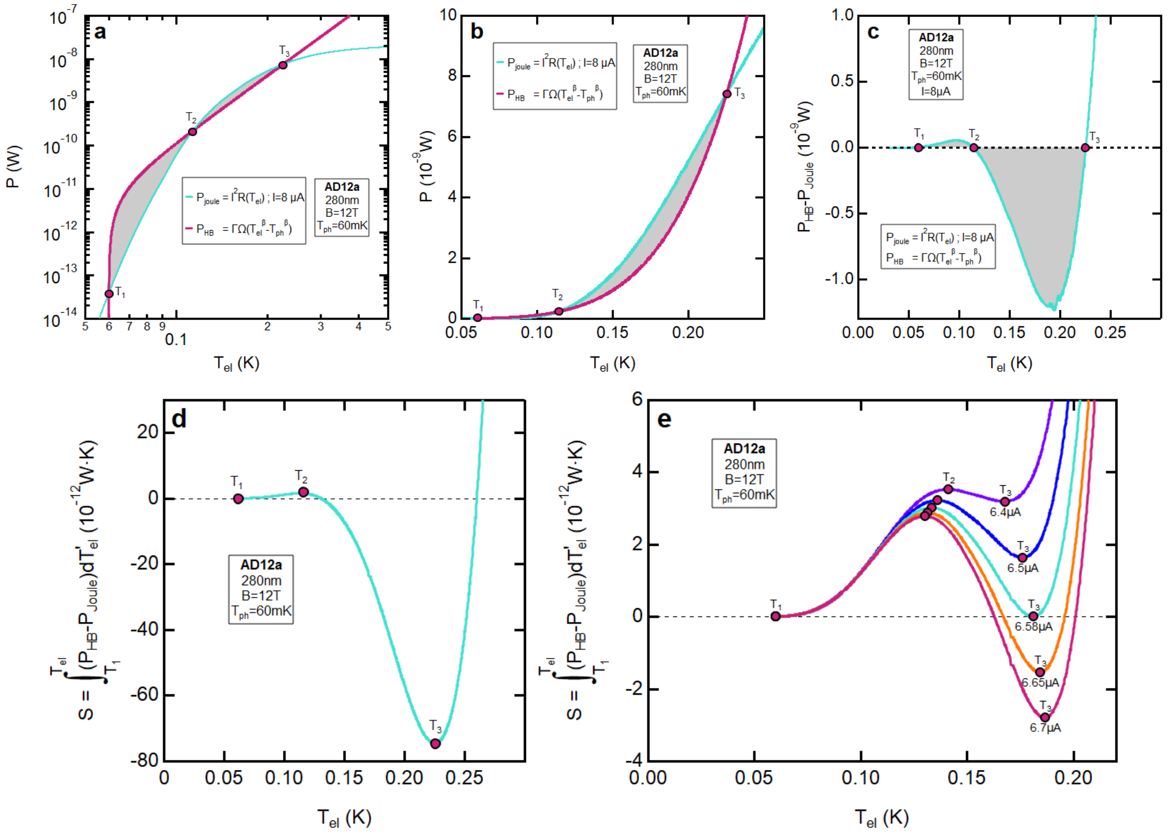

Supplementary Figure 8: **Extracting I_p from the equal area rule.** All figures describe the 280nm thick sample at B=12T and $T_{ph} = 60$mK. (a) $P_{HB} \equiv \Gamma\Omega(T_{el}^{\beta} - T_{ph}^{\beta})$ (magenta) and $P_{Joule} = I^2R(T_{el})$ (turquoise) vs. T_{el} plotted on a log-log scale for $I = 8\mu$A. We mark the two stable solutions as T_1 and T_3 and the unstable solution as T_2. The shaded regions mark the trapped area between the two curves. (b) Here we plot the same data as in (a) on a linear scale. (c) $P_{HB} - P_{Joule}$ from Figure (b) vs. T_{el}. (d) $S(T_{el}, I) \equiv \int_{T_1}^{T_{el}} (P_{HB} - P_{Joule})dT'_{el}$, which is the integrated area between the curves from T_1 up to some T_{el} vs. T_{el}. I_p that satisfies the equal area rule is found by the condition $S(T_3, I_p) = 0$. (e) $S(T_{el}, I)$ vs. T_{el} plotted for different values of I between 6.4μA and 6.7μA. It can be seen that $S(T_3, 6.58\mu\text{A}) = 0$ therefore $I_p = 6.58\mu$A.

vanish. I_p is found using an equal area rule (similar to Maxwell's equal area law in thermodynamics) from the condition $S(T_3, I_p) \equiv \int_{T_1}^{T_3} (P_{HB} - P_{Joule})\kappa(T_{el})dT_{el} = 0$ where κ is the thermal conductivity, $P_{HB} \equiv \Gamma\Omega(T_{el}^{\beta} - T_{ph}^{\beta})$ and $P_{Joule} = I^2R(T_{el})$.

In Supplementary Figure 8 below we follow this analysis and show that it is consistent with our experimental results, predicting only a very limited hysteresis where $I_p \approx I_c^{min}$. For simplicity we follow Ref. [25] and assume that κ does not vary significantly with T_{el} which simplifies the equal area rule to Supplementary Equation (7).

$$S(T_3, I_p) \equiv \int_{T_1}^{T_3} (P_{HB} - P_{Joule})dT_{el} = 0 \tag{7}$$

In Supplementary Figure 8a we plot P_{HB} (magenta) and P_{Joule} (turquoise) vs. T_{el} on a log-log scale for $I = 8\mu$A. We mark the two stable solutions as T_1 and T_3 and the unstable solution as T_2. The equal area rule (Supplementary Equation (7)) is satisfied if the two shaded areas in the figure are equal. Changing I will move the turquoise curve as a whole without changing its shape therefore increasing I will decrease the shaded area between T_1 and T_2 and increase the area between T_2 and T_3. From Supplementary Figure 8a it appears that the area between T_1 and T_2 is greater than the area between T_2 and T_3. This is actually an artifact of plotting this figure on a log-log scale. In Supplementary Figure 8b we plot the same data as in Supplementary Figure 8a but on a linear scale. It can be seen that the shaded area between T_2 and T_3 is actually much greater than the area between T_1 and T_2 therefore I_p that meets the equal area law should be smaller than 8μA. Note that here $I_c^{min} \approx 6.2\mu$A and $I_c^{max} \approx 41\mu$A therefore we

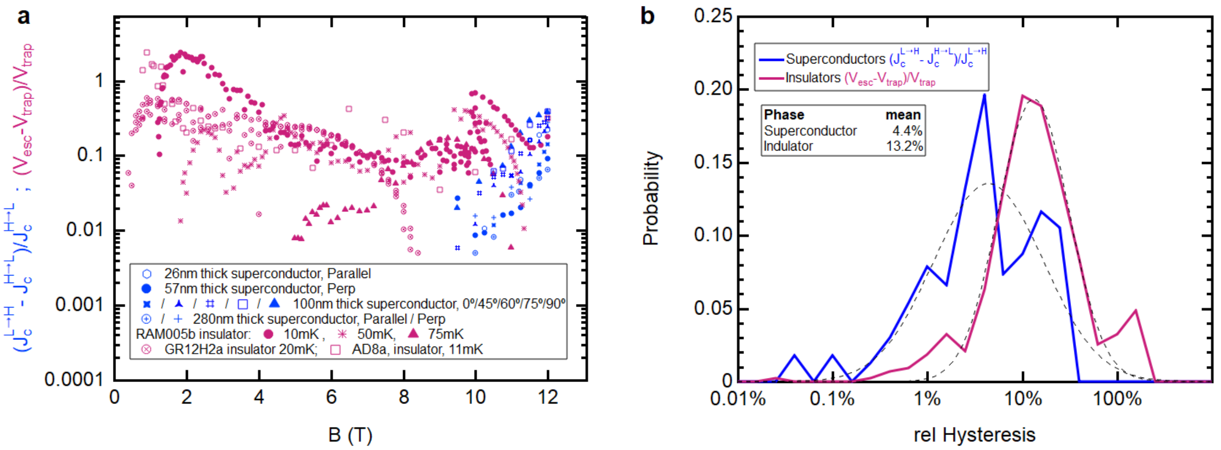

Supplementary Figure 9: **Relative hysteresis in the superconducting and insulating phases of a:InO.** (a) Relative hysteresis vs B. The blue data corresponds to $\frac{J_c^{L\to H}-J_c^{H\to L}}{J_c^{H\to L}}$ of the superconducting samples discussed in the main text. The magenta data marks $\frac{V_{esc}-V_{trap}}{V_{trap}}$ for a:InO samples in the insulating phase. The hysteresis was measured at various T's and B orientations as noted in the inset. (b) The distribution of relative hysteresis extracted from the data of (a) where the blue and magenta mark the recurrence of the relative hysteresis in the superconducting and insulating phases respectively. The dashed black lines mark log-normal fits to the data. The mean value of the relative hysteresis is noted in the inset.

see that $I_p \sim I_c^{min}$ which can account for the lack of hysteresis. In Supplementary Figure 8c we plot $P_{HB} - P_{Joule}$ from Supplementary Figure 8b vs. T_{el}. In Supplementary Figure 8d we plot $S(T_{el}, I) \equiv \int_{T_1}^{T_{el}} (P_{HB} - P_{Joule}) dT'_{el}$, which is the integrated area between the curves from T_1 up to some T_{el} vs. T_{el}. I_p that satisfies the equal area rule is found by the condition $S(T_3, I_p) = 0$ (as in Supplementary Equation (7)).

In Supplementary Figure 8e we plot $S(T_{el}, I)$ vs. T_{el} for different values of I between 6.4μA and 6.7μA. It can be seen that $S(T_3, 6.58\mu A) = 0$ therefore $I_p = 6.58\mu A$. In table. 2 we present the T_{ph} dependence of I_c^{max}, I_c^{min} and I_p. While I_c^{max} varies significantly with cooling, I_p hardly changes and remains similar to I_c^{min}.

T_{ph} [mK]	I_c^{max} [μA]	I_c^{min} [μA]	I_p [μA]
80	12	6.2	6.41
70	20	6.2	6.51
60	41	6.2	6.58
50	140	6.2	6.61
40	1100	6.2	6.63

Supplementary Table 2: ***T* dependence of theoretical I_c^{max}, I_c^{min} and I_p**

Supplementary Note 8. HYSTERESIS - COMPARING BETWEEN SUPERCONDUCTING AND INSULATING A:INO SAMPLES

In the main-text we noted that the LR→HR discontinuity is triggered prematurely, resulting in a limited hysteresis. Below we present a quantitative analysis of the hysteresis and compare it to the hysteresis measured in the insulating phase of a:InO.

We define the relative hysteresis in J_c as $\delta J \equiv \frac{J_c^{L\to H}-J_c^{H\to L}}{J_c^{H\to L}}$ and the relative hysteresis in insulating samples between V_c in the escape and trapping sides of the transition as $\delta V \equiv \frac{V_{esc}-V_{trap}}{V_{trap}}$. In Supplementary Figure 9a we plot δJ for superconducting samples in blue and δV for insulating samples in magenta. The relative hysteresis includes different B's, T's and B orientations relative to the sample's plane. In Supplementary Figure 9b we plot the distribution of relative hysteresis extracted from the data of Supplementary Figure 9a for superconducting (blue)

and insulating (magenta) samples. As the distribution of relative hysteresis is spread almost normally over several orders of magnitude we chose the bin-sizes in Supplementary Figure 9b to be logarithmically spaced (bin sizes of equal $\log(\delta J)$ and $\log(\delta V)$). The mean relative hysteresis (using a log-normal distribution) for the insulating samples we investigated is 13.2% and for superconducting samples it is 4.4%. While the relative hysteresis in the insulating phase is indeed three times larger than in the superconducting phase, for the time being we are unable to draw any conclusions from this difference. We note that the premature triggering of the escape transition is also observed in insulating a:InO samples and was previously interpreted as a result of the high disorder in the samples [7, 19, 24].

[1] Sacépé, B. et al. High-field termination of a cooper-pair insulator. Physical Review B **91**, 220508 (2015).
[2] Tinkham, M. Introduction to superconductivity (Courier Corporation, 2004).
[3] Boyd, R. Longitudinal critical current in type-ii superconductors. Physical Review **145**, 255 (1966).
[4] Misra, S., Urban, L., Kim, M., Sambandamurthy, G. & Yazdani, A. Measurements of the magnetic-field-tuned conductivity of disordered two-dimensional mo 43 ge 57 and ino x superconducting films: Evidence for a universal minimum superfluid response. Physical review letters **110**, 037002 (2013).
[5] Crane, R. et al. Survival of superconducting correlations across the two-dimensional superconductor-insulator transition: A finite-frequency study. Physical Review B **75**, 184530 (2007).
[6] Sacépé, B. et al. Low-temperature anomaly in disordered superconductors near b c2 as a vortex-glass property. Nature physics 1 (2018).
[7] Ovadia, M., Sacépé, B. & Shahar, D. Electron-phonon decoupling in disordered insulators. Physical review letters **102**, 176802 (2009).
[8] Golubkov, M. & Tsydynzhapov, G. Electron subsystem superheating as a cause of nonlinear current-voltage characteristics of amorphous ino x films. Journal of Experimental and Theoretical Physics Letters **71**, 516–519 (2000).
[9] Postolova, S. V., Mironov, A. Y. & Baturina, T. I. Nonequilibrium transport near the superconducting transition in tin films. JETP letters **100**, 635–641 (2015).
[10] Levinson, T., Doron, A., Tamir, I., Tewari, G. C. & Shahar, D. Direct determination of the temperature of overheated electrons in an insulator. Physical Review B **94**, 174204 (2016).
[11] Doron, A. et al. Nonequilibrium second-order phase transition in a cooper-pair insulator. Phys. Rev. Lett. **116**, 057001 (2016). URL http://link.aps.org/doi/10.1103/PhysRevLett.116.057001.
[12] Kapitza, P. The study of heat transfer in helium ii. J. Phys.(Moscow) **4**, 181 (1941).
[13] Johnson, R. C. & Little, W. Experiments on the kapitza resistance. Physical review **130**, 596 (1963).
[14] Pollack, G. L. Kapitza resistance. Reviews of Modern Physics **41**, 48 (1969).
[15] Pobell, F. Matter and methods at low temperatures, vol. 2 (Springer, 2007).
[16] Wellstood, F., Urbina, C. & Clarke, J. Hot-electron effects in metals. Physical Review B **49**, 5942 (1994).
[17] Lounasmaa, O. V. Experimental principles and methods below 1K (academic Press, 1974).
[18] Swartz, E. T. & Pohl, R. O. Thermal boundary resistance. Reviews of modern physics **61**, 605 (1989).
[19] Altshuler, B. L., Kravtsov, V. E., Lerner, I. V. & Aleiner, I. L. Jumps in current-voltage characteristics in disordered films. Phys. Rev. Lett. **102**, 176803 (2009).
[20] Bezuglyj, A. & Shklovskij, V. Effect of self-heating on flux flow instability in a superconductor near tc. Physica C: Superconductivity **202**, 234–242 (1992).
[21] Kunchur, M. N. & Knight, J. M. Hot-electron instability in superconductors. Modern Physics Letters B **17**, 549–558 (2003).
[22] Kunchur, M. N. Unstable flux flow due to heated electrons in superconducting films. Physical review letters **89**, 137005 (2002).
[23] Knight, J. M. & Kunchur, M. N. Energy relaxation at a hot-electron vortex instability. Physical Review B **74**, 064512 (2006).
[24] Doron, A. et al. Instability of insulators near quantum phase transitions. Physical review letters **119**, 247001 (2017).
[25] Gurevich, A. V. & Mints, R. Self-heating in normal metals and superconductors. Reviews of modern physics **59**, 941 (1987).
